# A DNA-based system for selecting and displaying the combined result of two input variables

**DOI:** 10.1038/ncomms10089

**Published:** 2015-12-08

**Authors:** Huajie Liu, Jianbang Wang, Shiping Song, Chunhai Fan, Kurt V. Gothelf

**Affiliations:** 1 Division of Physical Biology and Bioimaging Center, Shanghai Synchrotron Radiation Facility, CAS Key Laboratory of Interfacial Physics and Technology, Shanghai Institute of Applied Physics, Chinese Academy of Sciences, Shanghai 201800, China; 2 Department of Chemistry and iNANO, Center for DNA Nanotechnology, Aarhus University, Aarhus 8000, Denmark; 3 School of Life Science and Technology, ShanghaiTech University, Shanghai 200031, China

## Abstract

Oligonucleotide-based technologies for biosensing or bio-regulation produce huge amounts of rich high-dimensional information. There is a consequent need for flexible means to combine diverse pieces of such information to form useful derivative outputs, and to display those immediately. Here we demonstrate this capability in a DNA-based system that takes two input numbers, represented in DNA strands, and returns the result of their multiplication, writing this as a number in a display. Unlike a conventional calculator, this system operates by selecting the result from a library of solutions rather than through logic operations. The multiplicative example demonstrated here illustrates a much more general capability—to generate a unique output for any distinct pair of DNA inputs. The system thereby functions as a lookup table and could be a key component in future, more powerful data-processing systems for diagnostics and sensing.

## Abstract (web summary)

DNA based sensors provide substantial amounts of information that requires integration and processing. Here the authors demonstrate a DNA-based calculator that takes two inputs, identifies the solution in a library of answers and displays the result.

## Graphical abstract

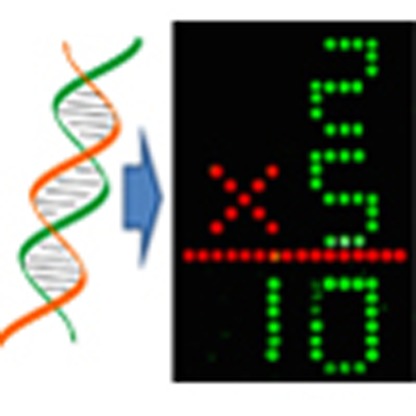

The power of DNA computing derives from its digital programmability and the parallel nature of the process by which vast numbers of interactions between populations of DNA molecules in solution occur in a short time frame^1^,^2^,^3^.

Current research in DNA computing has moved away from combinatorics, and instead primarily focused on processing inputs through logic operations. Numerous studies have explored DNA-based systems able to perform basic Boolean operations^4^. Impressive tile-based algorithmic computations^5^,^6^,^7^, strand displacement system^8^,^9^,^10^ and state machines^11^,^12^ have been reported. Furthermore, DNA and RNA have also been applied for implementing logic algorithms in cells^13^,^14^,^15^,^16^,^17^,^18^. The most complex system to date was reported by Qian and Winfree^19^: a four-bit circuit consisting of 130 strands that was capable of calculating the rounded of square root of numbers below 15, that is, the system applies four bits of input provides two bits of outputs. In an elegant design, Seelig and co-workers devised a strand displacement system that could be expressed *in vitro* from plasmids and process the input of two strands autonomously^20^.

Here we explore a different path showing how the combinatorial approach can also be applied as a practical means for processing molecular signals. The method is conceptually simple and experimentally straightforward, although not autonomous. In the first step, the system converts the input of two variables to a result, which is selected from a library of strands that match the combined inputs. In few other cases, the combined input of two strands has been applied to select a strand from a small library^21^,^22^,^23^. Rather than processing the input signals through a network of logic gates to calculate the solution, our system operates as a lookup table combining two arbitrary inputs to provide one output^24^ by selecting the correct result from a library of results of the system. To exemplify, we show that the system can find the result of the multiplication between two numbers.

The system we have devised, furthermore, converts the selected result to Arabic numerals with one or two digits that are displayed on different templates, from the nanoscale to the macroscale. With few exceptions, other DNA-computing networks provide the output in the form of optical signals such as absorption or emission, which significantly limits the number of outputs that can be differentiated in the same solution. In other examples the readout has been observed by mobility shifts in gels or by sequencing, which are slow and laborious methods. On other occasions the result of DNA logic operations has been readout using atomic force microscopy (AFM), showing single-nucleotide polymorphisms as a letter symbol on DNA origami^25^. In other work electrochemical readout has been applied for detection of the output of logic operations^26^. In our system the digit is formed by segregating the output value from the first selection in a set of sequences that will address the lines in a seven-segmented display that are required to write the result number. Recently, Poje *et al.*^27^ reported on a system that also provides the output of a DNA-based system in a macroscopic fluorescence-based seven-segmented display; however, in this system the input was based on Boolean logic gates. In our work we also show the readout of the seven-segmented display at the nanoscale on DNA origami and a display that can be read by the naked eye.

## Results

### The design principle

**Figure 1:**
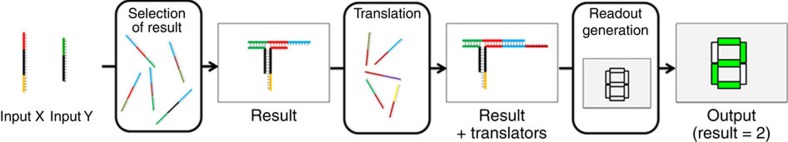
Illustration of the steps involved in the multiplication. Two input DNA strands X and Y (for example, 1 and 2) are converted into a digital presentation of the result.

The mechanism of the DNA-based system is outlined in more detail in Fig. 1. The two input strands X and Y each represent a number. The red and green parts of the input sequences contain the information that is specific for the number they represent. These domains are each 8-nt long. The black part of the sequences (16 nt) is universal so that all input X sequences can form a hybrid with all Y sequences. The yellow part of input X is a capture sequence that is used for separation. When the input strands are mixed with a library of result strands the input strands will form a stable three-way junction result with, and only with, the fully complementary result strand containing two domains that are complementary to the red and green parts of the input strands. The system may be viewed as a type of AND gate since both X and Y have to be present to select the result; however, it is not binary since X and Y may in principle each represent up to 2^8^ different values. The selective binding of the combined input to one specific result strand in the result library is the key element of the method. The result strand contains a blue extension with a unique sequence representing the value that corresponds to the multiplication of X and Y. Next, the value is translated into a mixture of result–translator complexes by incubation with a library of translator strands. If, for example, the result of the calculation is 2 then the translator strands that represent 2 will be selected. For the presentation of the number 2 in a seven-segmented display, five out of seven lines must be addressed and, that is, five different translator sequences are employed to display the digit 2. Hence, the result strand representing the number 2 will hybridize with a mixture of the translator strands representing 2. In the final readout generation the result–translator complexes are separated from the translator library and subjected to a universal display platform on a surface where the translator strands bind to the domains of the digit that corresponds to the result as illustrated for the result 2 in Fig. 1. We demonstrated the readout on three different platforms: two macroscopic displays that provide a fluorescent and a visual readout, and on the nanoscale on a DNA origami platform where the readout is imaged using AFM.

### Analysis of the selection processes

**Figure 2:**
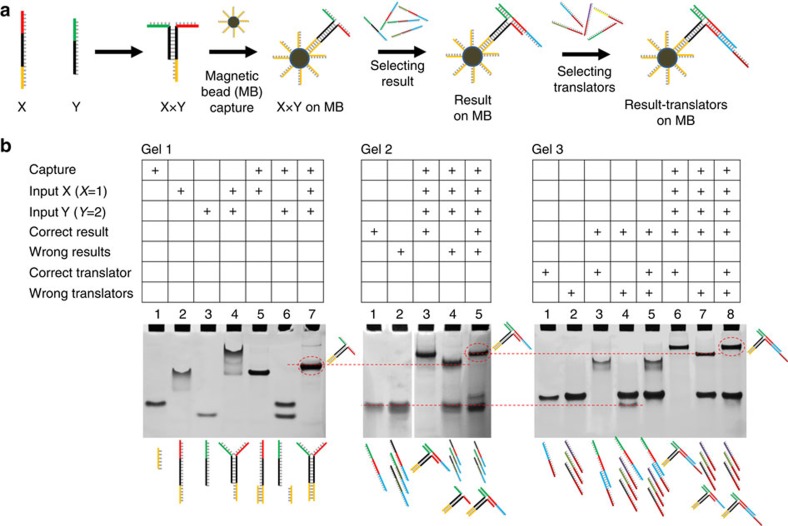
Illustration of the steps involved in the multiplication. (**a**) Illustration of the steps involved in the process from the input hybridization, capture on MB, result selection and translator selection. (**b**) Native PAGE analysis of the hybridization processes involved in the process.

The hybridization interactions involved in the result selection and translation processes were analysed using polyacrylamide gel electrophoresis (PAGE). All hybridization events involved in the selection and translation, including capture on magnetic beads (MBs), are shown in Fig. 2a. The MBs are used for two separation steps: removal of the solution containing the library of result strands after the first selection and removal of the solution containing the translator strands after the translation. The MBs are omitted in the gel analysis experiments (Fig. 2b).

First, hybridization of the input strands was analysed using native PAGE as shown in Fig. 2b Gel 1. The DNA strands representing the multiplication of 1 × 2 (X1, Y2) were used as an example. In addition to the two input strands, a short capture strand that has the same sequence as the capture strand on the MB was eluded in lanes 1–4. It is observed that X1 tails in the gel (lane 2). Sequence analysis revealed that X1 could form a weak dimer. However, it is avoided by hybridization to the capture strand (lane 5). The gel results clearly show that the two inputs X1 and Y2 binds to each other (lane 4). The mobility of the X1, Y2 duplex is too low and it tails. Sequence analysis revealed that X1 and Y2 could form a weak dimer. However, this is avoided by hybridization to the capture strand (lane 7).

The selection of the correct result strand from a small library of four result strands is shown in Fig. 2b Gel 2. In lanes 3–5 the interaction with the correct result, the wrong result and a mixture of the two is shown, and only in the presence of the correct result a band with lower mobility is observed. It should be noted that the wrong results have 8-nt domains that are complementary to the input; however, only the three-way junction formed with the fully matching (16 nt) result is expected to be stable. The selection of the correct result strand from a full library of 15 results is shown in Supplementary Fig. 1.

The hybridization interactions involved in the translation are shown in Gel 3. Here a translator library of four strands is applied, but it was also verified for full libraries (Supplementary Fig. 2). In lanes 6–8 it is confirmed that only the correct translator is selected and it appears as the slower migrating band in lanes 6 and 8. In the gel examples the use of MBs was omitted and no separation was performed. However, we have also shown with PAGE that the complexes were liberated from the MBs by incubation in pure water (Supplementary Fig. 3).

**Figure 3:**
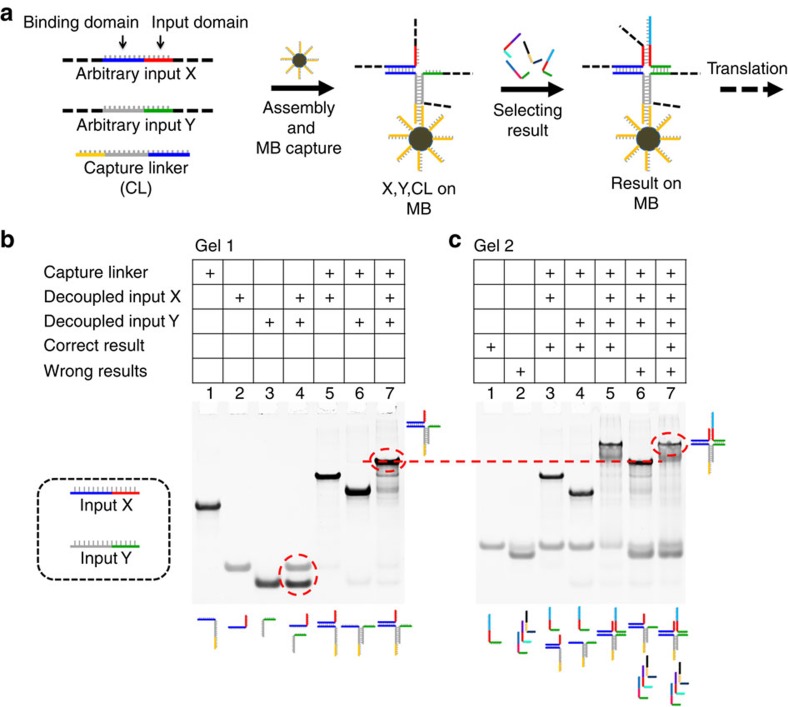
Selection of a result from arbitrary input sequences X and Y. (**a**) Illustration of the steps involved in the process from the input hybridization with the capture–linker and MBs, and result selection. (**b**) Native PAGE analysis of the hybridization processes involved in the formation of the input complex. (**c**) Native PAGE analysis of the hybridization processes involved in the selection of the correct result strand in a four-way junction.

If the method should be used to process the input of two biological sequences it would require that X and Y be decoupled to allow input of arbitrary sequences. For this purpose we have introduced a third capture–linker strand as shown in Fig. 3a. This sequence contains two 8-nt domains that are each complementary to the two inputs, and furthermore it contains the capture sequence that allows binding to the MBs. This forms an incomplete four-way junction that is completed by selecting the fully complementary result sequence on the two 8-nt domains on X and Y to form a stable four-way junction. To demonstrate the principle we have used two short synthetic input strands. As shown with native PAGE in Fig. 3b, lane 4, inputs X and Y are independent. In lane 7, inputs X, Y and the capture–linker form a stable complex. In the native PAGE result shown in Fig. 3c, lanes 3 and 4, neither X nor Y alone binds with the result strand. In lane 5, where both X and Y are present, the input complex can bind to the correct result. This means that the correct result is only selected in the presence of both inputs X and Y. In lane 6 it is demonstrated, using three results where only one of the domains is correct, that wrong results do not bind to the input complex. Finally, in lane 7 the correct result is selected in the mixture with three wrong result sequences.

### Translation of the results to a seven-segmented display

**Figure 4:**
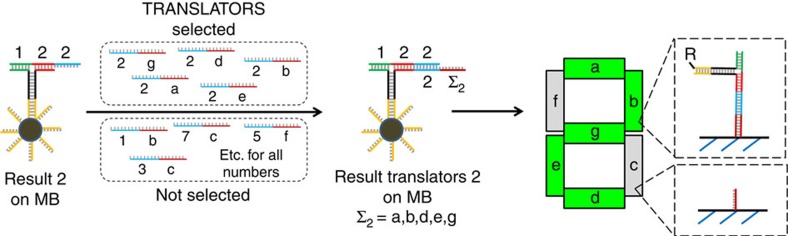
Illustration of the selection of translator strands and immobilization on a seven-segmented display. The result `2' complex captured on magnetic beads is mixed with the translator library resulting in selection of the strands required to display 2 in the seven-segmented display. R: Contrast agent for imaging such as a dye or magnetic bead.

The mechanism of the output generation proceeds by selection of translator strands that guide the assembled complex to the lines of the digital number that displays the result in a seven-segmented display. As illustrated in Fig. 4 for the simple calculation of 1 × 2=2, the result two strands can hybridize with each of the five translator strands that are required to display the number 2. After MB-mediated separation from the solution of translator strands, the result–translator complex is (in most cases) liberated from the MBs and applied to a surface containing a pattern of one or more digital numbers. Each of the lines in the digit is functionalized with a unique host sequence that is complementary to the translator stands a–g. This means that contingent on the selected set of translator strands the digit has the capacity to display all numbers from 0 to 9. The encoding required for displaying the numbers 0–9 is shown in Table 1. A total of 49 translator sequences are required for displaying the 10 numbers in a one-digit display.

### Displaying of results in three different displays

**Figure 5:**
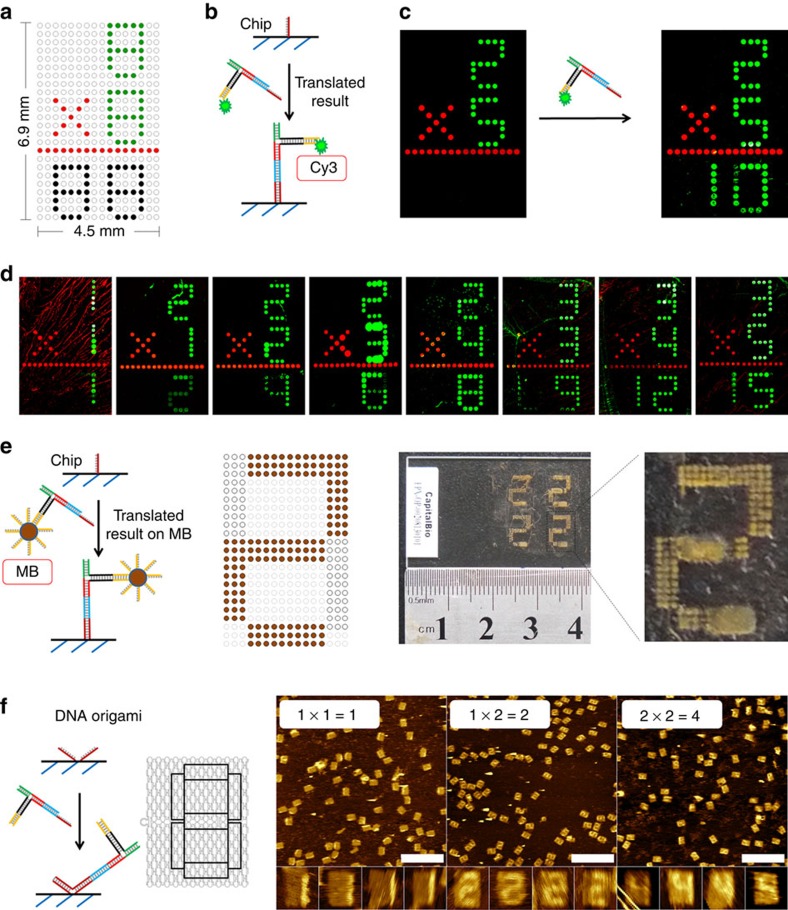
Displaying the results of multiplication reactions on seven-segmented displays. (**a**) The design of the first DNA chip used to display the results. The values of inputs X and Y are printed with Cy3, an operator of ‘ × ' and a line are printed with Cy5-labelled strands. Host strands are printed on the output area for digital display of calculated results. (**b**) The recognition between host and translated result. (**c**) Fluorescent images by a plate reader of the chip before and after the result–translator complex was added. (**d**) The results of a series of multiplications imaged by a plate reader. (**e**) Displaying the result by immobilization of the result–translator complex connected to MBs on a DNA chip. The right-hand panel shows a photo and its magnification of the result of the 1 × 2 multiplication. (**f**) Immobilization of the unlabelled result–translator complex on V-shaped patterns on DNA origami and imaging of the output numbers of three multiplications using AFM. Scale bars, 500 nm.

Digital displaying of the result was demonstrated using three different platforms. First, a two-digit readout was realized on a DNA chip (DNA microarray) for millimetre-scale digital output. The DNA chip is prepared on an epoxy-coated slide by spotting selected amino-modified host DNA sequences into the desired patterns: here in a typical square lattice pixelated design (Fig. 5a). Four of the same patterns were printed on one slide and each pattern displays a multiplication formula with a size of 4.5 mm × 6.9 mm (Fig. 5a and Supplementary Fig. 4). The input digits, multiplication sign and a line are preprinted with Cy3- and Cy5-labelled DNA, respectively. The bottom two result digits are functionalized with host strands that could recognize the translated result through translator–host hybridization (Fig. 5b and Supplementary Fig. 5a). To provide a fluorescent signal the input X is functionalized with a Cy3 dye, which will remain in the result complex after separation from the MBs.

With this set-up we have performed 15 calculations with inputs X and Y ranging from 1 to 5 and results ranging from 1 to 15. The library of results for each calculation was a reduced library of four result members where one result is correct. To guide the result complex to both numbers of a two-digit result, a library of result strands that addresses both digits is applied and the details of the translator library and display mechanism can be found in Supplementary Fig. 6. Using 2 × 5 as an example of a multiplication, the inputs 2 and 5 were preprinted on the chip and imaged by a microarray scanner (Fig. 5c). The calculation of 2 × 5 was performed as described above by selecting first the result strand and then the translator strands. After separation from the MBs a solution of the result complex was added to the chip. After incubating for 2 h at room temperature (RT) followed by washing and drying the fluorescence image of the chip displayed the result ‘10' in the two-digit seven-segmented display.

A series of other multiplications were performed in a similar manner but with other inputs, and the correct results are shown in Fig. 5d. However, some calculations did not show a result or the result was defect (Supplementary Figs 7–9). Out of a total of 15 multiplications 11 were correctly displayed, whereas 4 were wrong (Supplementary Fig. 10). To further analyse the fidelity of the method, we tested the readout for all combinations of inputs (Supplementary Figs 11 and 12 and Supplementary Table 1). The minimum limit of detection of the result complex for this assay was detected to be 10 nM (Supplementary Fig. 13)

By a slight change in the chip design it can also provide the output in a form that is visual to the naked eye. The MBs have a characteristic brown colour, and it appeared that if the result complex that is immobilized on the MB is directly applied to the chip, the digit lines of the correct result appears as brown number patterns (Fig. 5e). The size of the pattern was increased to provide a result that can be read without magnification. Photographs of the display of the result 2 of the multiplication 1 × 2 is shown in the right panels of Fig. 5e. This procedure is more simple and efficient since cleavage from the MBs is evaded and since no fluorophore and imaging equipment is required.

In the third example, one-digit results of multiplications were displayed at the nanoscale on a DNA origami template and the numbers were imaged with AFM. DNA origami is a self-assembled DNA nanostructure that consists of parallel aligned DNA helices that are interlinked by crossover of DNA strands^28^,^29^. It is formed from a >7,000-nt-long single-stranded scaffold and >200 short synthetic staple strands. Single-stranded extensions of staple strands can be positioned at unique sites at the origami surface and be used for immobilization of the translator strands by specific hybridization. We used the V-shaped binding domain reported by Yan and co-workers for optimal binding to the origami surface (Fig. 5f and Supplementary Fig. 5b)^30^. The ‘8'-shaped patterns were designed on the origami containing at least 6V-shaped host sites for each of the seven lines of the digit (Supplementary Fig. 14). The steric bulk of the result complex provides sufficient contrast to allow imaging by AFM of the line of the digits to which the result complex is bound. As shown in Fig. 5f, the results of calculations 1 × 1, 1 × 2 and 2 × 2 display correctly at the surface, and the yields of correctly displayed results in the AFM images have been determined to be 53%, 72% and 25%, respectively (Supplementary Fig. 15).

In a much-simplified version of the readout mechanism, the DNA strands in the result library were immobilized in separate spots on a DNA chip (Supplementary Fig. 16). This should allow the combined inputs, of which one is conjugated to a fluorophore, to bind to the spot containing the fully complementary strand. The location of that spot would then indicate the result and no translation would be required. When we tested this approach some correct results were observed; however, it was found to be less specific (Supplementary Fig. 17). The binding of the combined result strands in a three-way junction at the chip surface provided false positives and false negatives. This may be caused by a weaker binding efficiency at the surface because of the charge interactions and steric bulk of the three-way junction. Furthermore, the physical separation of the result strands at the chip eliminates the competition between the result strands and therefore increases the nonspecific binding.

## Discussion

In this work we have demonstrated examples of a DNA-based lookup table exemplified by the selection of results of a multiplication. It should, however, be clear that we could have chosen any operation that requires two inputs and results in a one- or two-digit output. Since the method can differentiate the order of the inputs, that is, sequence-wise 1 × 2 is different from 2 × 1, the method could also be used for subtraction. In more general terms, it is a method to retrieve a *relation* between individuals in populations X and Y that has been encoded in the result library. It would also be possible for one combination of inputs X and Y to have selected more results. Since each of inputs X and Y are encoded by 8 nt, the input libraries can principally have 65,536 different members each and the result library can principally have 4.29 billion members. Synthesizing this number of sequences is obviously unrealistic, and cross-reactions between sequences and self-complementarity would be an increasing problem the closer the library numbers come to the principal limits. However, the method has sufficient diversity to cover synthetically realistic numbers of inputs and results.

The translation is also a selection process; however, in contrast to the result selection where two inputs are converted into one result, the translation segregates the result in an array of data that are required for displaying the result. We have demonstrated this concept for the seven-segmented displays, and the mechanism by which the result is translated resembles an electronic calculator. In the translation the result is converted into a binary code that addresses the individual lines of the display (Table 1).

Most of DNA-based logic systems are binary and thus also require binary inputs where a DNA strand represents 0 or 1. The translation process described here would enable the conversion of higher-order information in a DNA input strand to an array of binary numbers.

As the complexity of oligonucleotide-based technologies such as biosensing and regulating systems in synthetic biology increases, more complex and multiplexed output information of such systems becomes available. The method presented here is an example of a method to combine pieces of information encoded in DNA strands and process it to a common result that is displayed in an immediately readable format. For example, two disease-marker oligonucleotide inputs of biologic origin could potentially be combined on a template via the four-way junction method and only if both markers were present they would select a solution from a solution library.

## Methods

### Materials

All short DNA strands were purchased from Invitrogen (China) and Shanghai Sangong Biotech and used as received. M13mp18 single-stranded DNA was purchased from New England Biolabs. Chemicals were purchased from Sinopharm and Sigma-Aldrich. Epoxy group-coated slides were bought from CapitalBio Corporation (product name: OPEpoxySlide). Streptavidin-coated magnetic microbeads were bought from Invitrogen (product name: Dynabeads MyOne STV C1).

### Multiplication on MBs

For a typical multiplication, 30 μl of MBs (10 mg^−1 ^ml) from the supplier were placed on a magnet to remove the supernatant with a pipette and re-dispersed in 60 μl of 1 × TAE-Mg buffer (20 mM Tris, pH 7.6, 2 mM EDTA, 12.5 mM MgCl_2_) to a final concentration of 5 mg^−1 ^ml. These MBs were mixed with the capture strand (25 μl, 50 μM in 1 × TAE-Mg) at RT for 30 min to immobilize it on MBs. The excess and weakly absorbed strands were removed through magnet-assisted washing of the MBs for two times with 1X TAE-Mg. The capture-immobilized MBs were resuspended in 60 μl of 1 × TAE-Mg. A solution of input X and input Y in 1 × TAE-Mg (60 μl, 20 μM for input X and 30 μM for input Y) was then added to the capture-immobilized MBs and to incubate this mixture at 4 °C for 30 min. The unbound DNA was removed with magnet-assisted washing and the X × Y on MB complexes were re-dispersed in 40 μl of 1 × TAE-Mg. This sample was then incubated with a mixture of one correct result strand and some wrong result strands (60 μl, 20 μM for each strand) at 4 °C for 30 min. Remove the unbound DNA with magnet-assisted washing and resuspend this sample in 40 μl of 1 × TAE-Mg. Next, the sample was incubated with a mixture of correct and wrong translator strands (60 μl, 20 μM for each strand) at 4 °C for 30 min. After magnet-assisted washing, the supernatant was discarded and the pellet was re-dispersed and incubated in heated deionized water (18 μl, 60 °C) for 30 min. This mixture was then placed on a magnet and the 18 μl of the supernatant was collected. Finally, 2 μl of 10 × TAE-Mg was added to the collected solution and the result–translator complexes could be obtained after slowly cooling it to RT. For PAGE analysis, samples were loaded on a 15% native gel containing 1 × TBE and 12.5 mM Mg^2+^. Run the gel at 50 V, 4 °C for 4 h and stain it with Stains-All or ethidium bromide. All gels were repeated at least two times.

### Display on DNA chip

An ArrayIt SpotBot 2 microarray spotter was used for the preparation of addressable millimetre-scale patterns on an epoxy-coated slide. First, according to the design of each pattern, a corresponding printing programme was made using the Multiple Microarray Format SpoCLe Generator (ArrayIt). Second, DNA solutions (10 μM in 1 × ArrayIt's Micro Spotting Solution, placed in a 384-well plate) were spotted, using a 946MP3 Micro Spotting Pin, on the slides to form the patterns. Third, after incubating the slides at 37 °C overnight, the unprinted areas of the slides were blocked using a solution of 0.01% BSA, 0.1% SDS, 5 × SSC buffer at RT for 1 h. These slides were then washed with 0.1 × SSC buffer for three times and 5 min each. Fourth, a solution of a translated result (1 μM in 1 × TAE-Mg) was added on its corresponding slides for hybridizing with its Host strands. Fifth, after incubating at RT and in dark for 2 h, the solution on the slides was discarded and the slides were washed with three washing solutions sequentially (washing solution1: 2 × SSC, 0.2% SDS, 10 min; washing solution2: 2 × SSC, 5 min; washing solution3: 0.2 × SSC, 0.5 min). Finally, the slide surface was dried with nitrogen flow and the calculated results displayed on the slides were read out with a GenePix 4100A Microarray Scanner.

### Display on DNA origami

For preparing DNA origami short DNA staple strands and the M13mp18 scaffold strand were mixed in 1 × TAE-Mg buffer with the final concentrations of 100 and 5 nM, respectively. The mixture was annealed from 95 to 5 °C (1 °C min^−1^) using a PTC-200 Peltier Thermal Cycler (MJ Research)^28^. Directly deposit the DNA origami solution (5 μl) on a freshly cleaved mica surface and leave it to absorb for 5 min. The mica surface was washed with 1 × TAE-Mg for two times and incubated with a solution of the translated result at RT for 30 min. AFM was used to display the calculated results on DNA origami through scanning with a Bruker Multimode Nanoscope VIII instrument under tapping mode in fluid with SNL-10 tips (Bruker).

## Additional information

**How to cite this article:** Liu, H. *et al.* A DNA-based system for selecting and displaying the combined result of two input variables. *Nat. Commun.* 6:10089 doi: 10.1038/ncomms10089 (2015).

## Supplementary Material

Supplementary Information Supplementary Figures 1-17 and Supplementary Table 1.

Supplementary Data 1 List of DNA sequences.

## Figures and Tables

**Table 1:** Encoding for displaying the numbers 0–9 in a seven-segmented display.

**Digit**	**a**	**b**	**c**	**d**	**e**	**f**	**g**
0	1	1	1	1	1	1	0
1	0	1	1	0	0	0	0
*2*	*1*	*1*	*0*	*1*	*1*	*0*	*1*
3	1	1	1	1	0	0	1
4	0	1	1	0	0	1	1
5	1	0	1	1	0	1	1
6	1	0	1	1	1	1	1
7	1	1	1	0	0	0	0
8	1	1	1	1	1	1	1
9	1	1	1	1	0	1	1

Translator strands required for displaying the number 2 are shown in italics. 1, translator sequence present for the corresponding digit; 0, translator sequence not present for the corresponding digit.
