# Supplementary material for: A DNA-based system for selecting and displaying the combined result of two input variables
Source: Nat Commun. 2015 Dec 8;6:10089. doi: 10.1038/ncomms10089 (PMC4686758; doi:10.1038/ncomms10089)
Supplement: Supplementary Information — Supplementary Figures 1-17 and Supplementary Table 1. [file ncomms10089-s1.pdf]

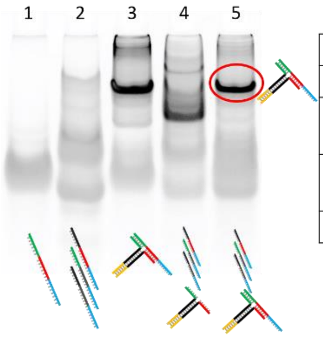

Lane 1	1 correct result
Lane 2	14 wrong results
Lane 3	inputs + 1 correct result
Lane 4	inputs + 14 wrong results
Lane 5	inputs + all results

Supplementary Figure 1. PAGE analysis of the selection of the correct result strand from the full library.

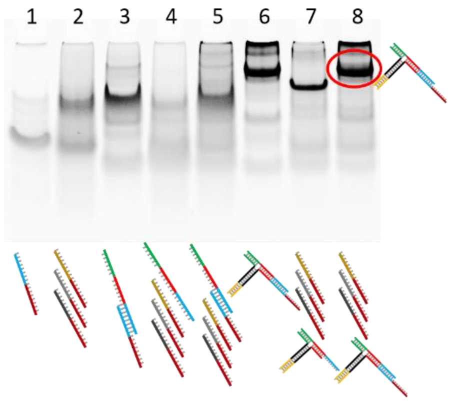

Lane 1	1 correct translator
Lane 2	47 wrong translators
Lane 3	result + 1 correct translator
Lane 4	result + 47 wrong translators
Lane 5	result + all translators
Lane 6	inputs-result complex + 1 correct translator
Lane 7	inputs-result complex + 47 correct translators
Lane 8	inputs-result complex + all translators

Supplementary Figure 2. PAGE analysis of the selection of the correct translator strand from the full library.

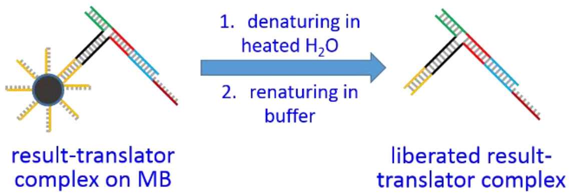

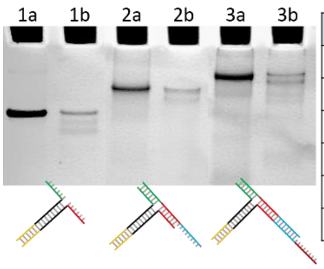

lane	sample	
1a	preformed	inputs
1b	released	
2a	preformed	inputs + result
2b	released	
3a	preformed	inputs + result + translator
3b	released	

Supplementary Figure 3. Illustration and PAGE analysis of the releasing of result-translator complexes from MBs.

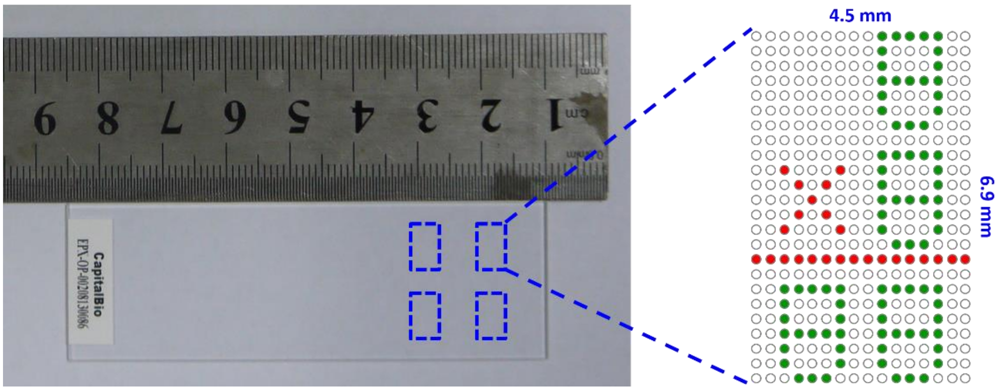

Supplementary Figure 4. A photograph of the epoxy-slide with the positions of the printed areas.

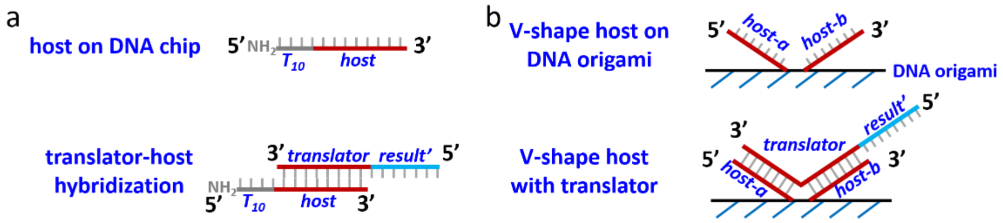

Supplementary Figure 5. Host strands used for display on DNA chip (a) and DNA origami (b).

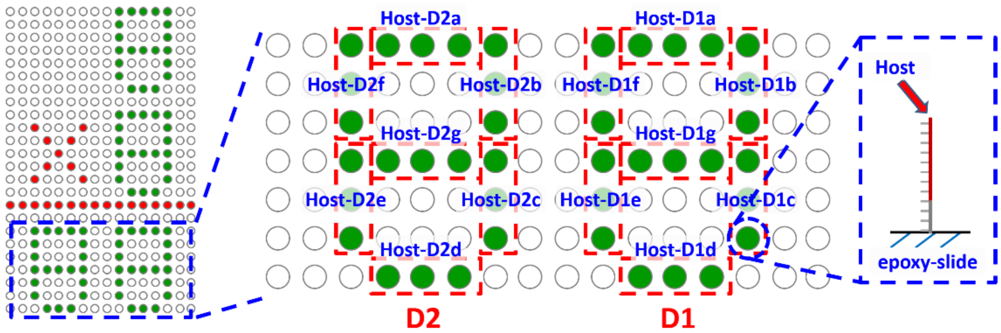

value of X	value of Y	value of X×Y	Result strand	Translator strands	Host strands
1	1	1	Result11	Translator1D1b, 1D1c	Host-D1b, D1c
1	2	2	Result12	Translator2D1a, 2D1b, 2D1d, 2D1e, 2D1g	Host-D1a, D1b, D1d, D1e, D1g
1	3	3	Result13	Translator3D1a, 3D1b, 3D1c, 3D1d, 3D1g	Host-D1a, D1b, D1c, D1d, D1g
1	4	4	Result14	Translator4D1b, 4D1c, 4D1f, 4D1g	Host-D1b, D1c, D1f, D1g
1	5	5	Result15	Translator5D1a, 5D1c, 5D1d, 5D1f, 5D1g	Host-D1a, D1c, D1d, D1f, D1g
2	1	2	Result21	Translator2D1a, 2D1b, 2D1d, 2D1e, 2D1g	Host-D1a, D1b, D1d, D1e, D1g
2	2	4	Result22	Translator4D1b, 4D1c, 4D1f, 4D1g	Host-D1b, D1c, D1f, D1g
2	3	6	Result23	Translator6D1a, 6D1c, 6D1d, 6D1e, 6D1f, 6D1g	Host-D1a, D1c, D1d, D1e, D1f, D1g
2	4	8	Result24	Translator8D1a, 8D1b, 8D1c, 8D1d, 8D1e, 8D1f, 8D1g	Host-D1a, D1b, D1c, D1d, D1e, D1f, D1g
2	5	10	Result25	Translator0D1a, 0D1b, 0D1c, 0D1d, 0D1e, 0D1f, 1D2b, 1D2c	Host-D1a, D1b, D1c, D1d, D1e, D1f, D2b, D2c
3	1	3	Result31	Translator3D1a, 3D1b, 3D1c, 3D1d, 3D1g	Host-D1a, D1b, D1c, D1d, D1g
3	2	6	Result32	Translator6D1a, 6D1c, 6D1d, 6D1e, 6D1f, 6D1g	Host-D1a, D1c, D1d, D1e, D1f, D1g
3	3	9	Result33	Translator9D1a, 9D1b, 9D1c, 9D1d, 9D1f, 9D1g	Host-D1a, D1b, D1c, D1d, D1f, D1g
3	4	12	Result34	Translator2D1a, 2D1b, 2D1d, 2D1e, 2D1g, 1D2b, 1D2c	Host-D1a, D1b, D1d, D1e, D1g, D2b, D2c
3	5	15	Result35	Translator5D1a, 5D1c, 5D1d, 5D1f, 5D1g, 1D2b, 1D2c	Host-D1a, D1c, D1d, D1f, D1g, D2b, D2c

Supplementary Figure 6. Detailed digital display mechanism on DNA chip. According to the design, a specific Host strand is printed at its corresponding areas shown in this figure. For each specific calculation, X×Y could only recognize its corresponding result and translator strands. Then, the specific translator strand could recognize and bind to its corresponding host strand. The specific result, translator and host strands for each multiplication are shown in the table.

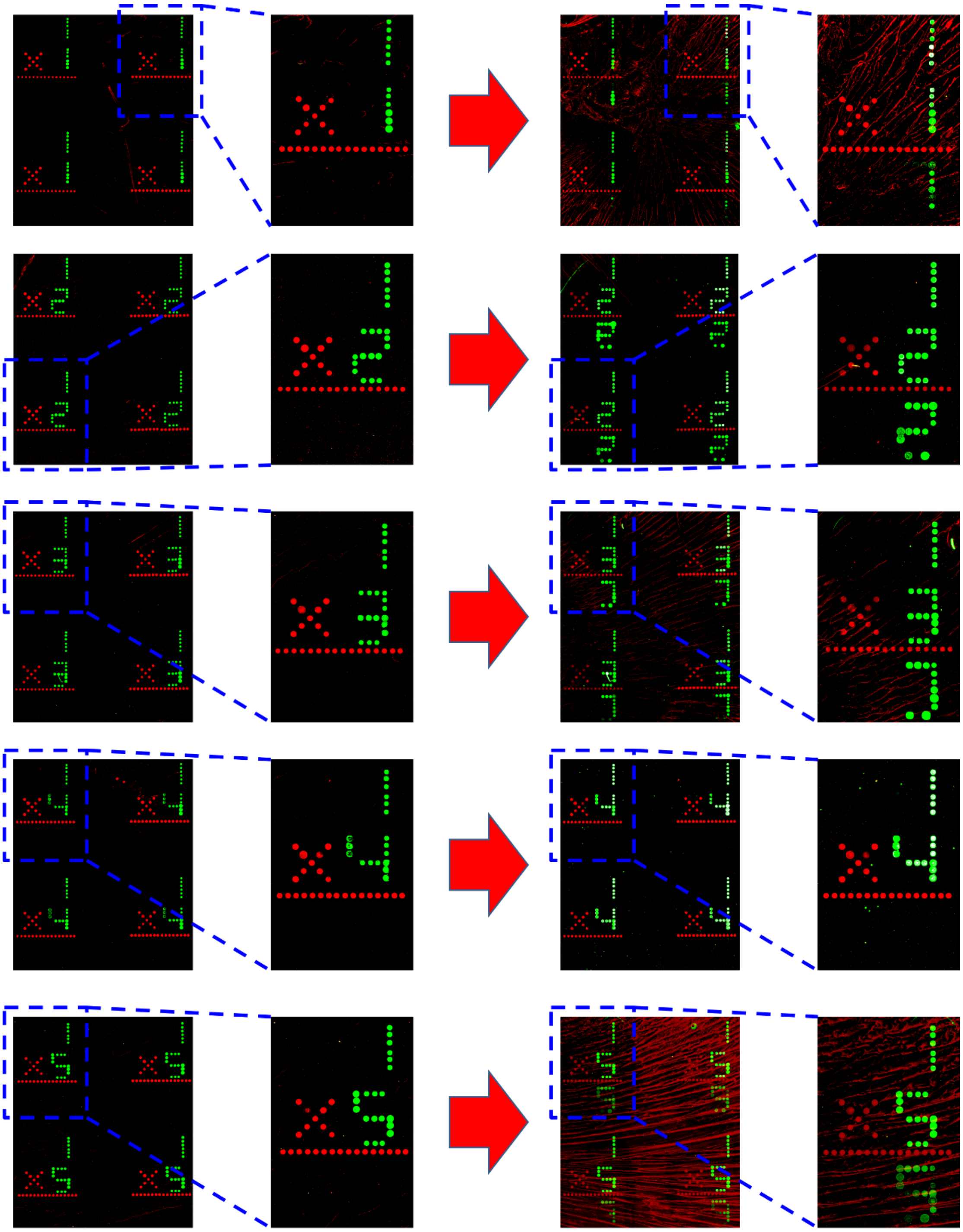

Supplementary Figure 7. Complete showing of “1×n” (n=1, 2, 3, 4, 5) on DNA chips.

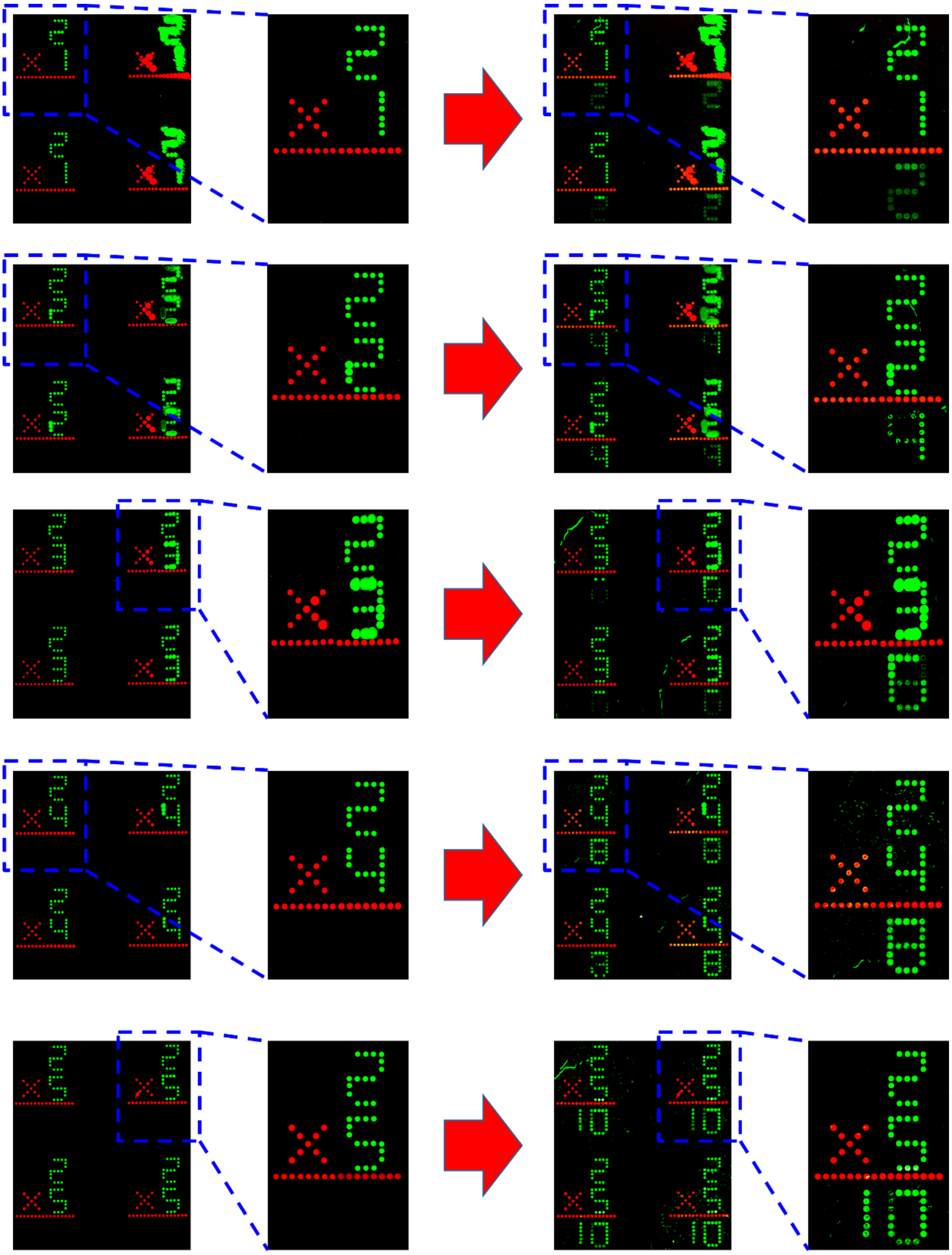

Supplementary Figure 8. Complete showing of “2×n” (n=1, 2, 3, 4, 5) on DNA chips.

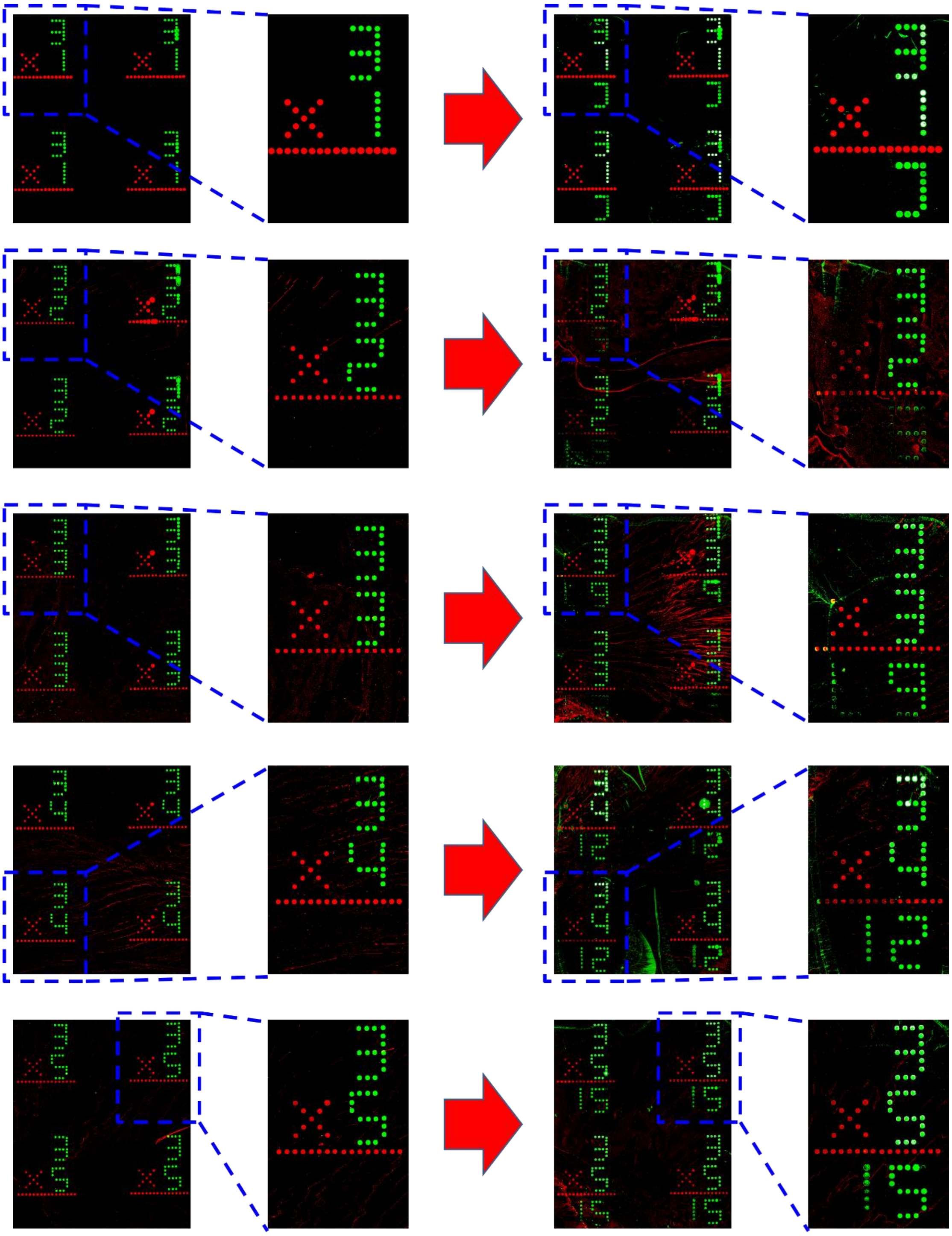

Supplementary Figure 9. Complete showing of “3×n” (n=1, 2, 3, 4, 5) on DNA chips.

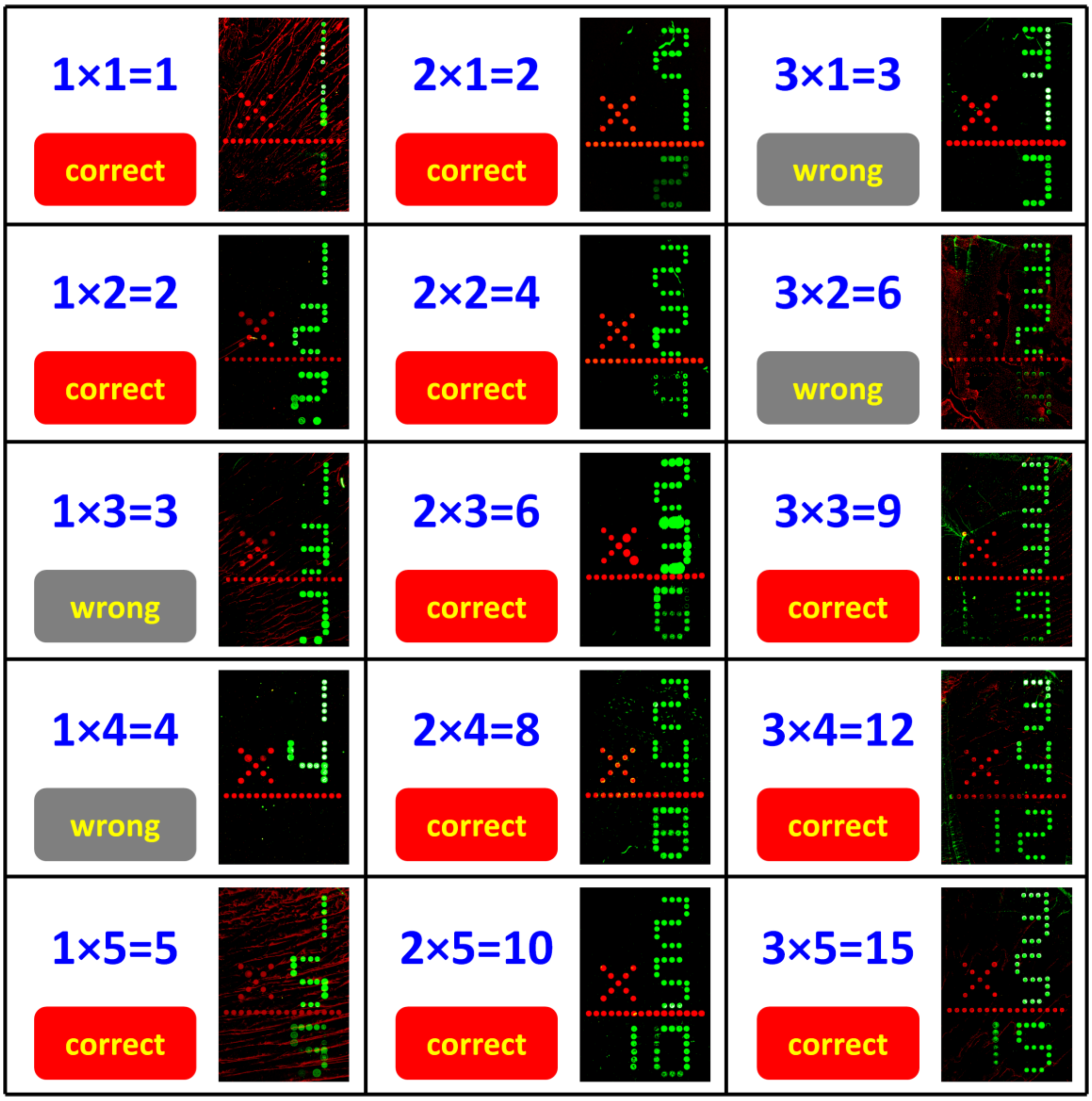

Supplementary Figure 10. All multiplications displayed on DNA chips.

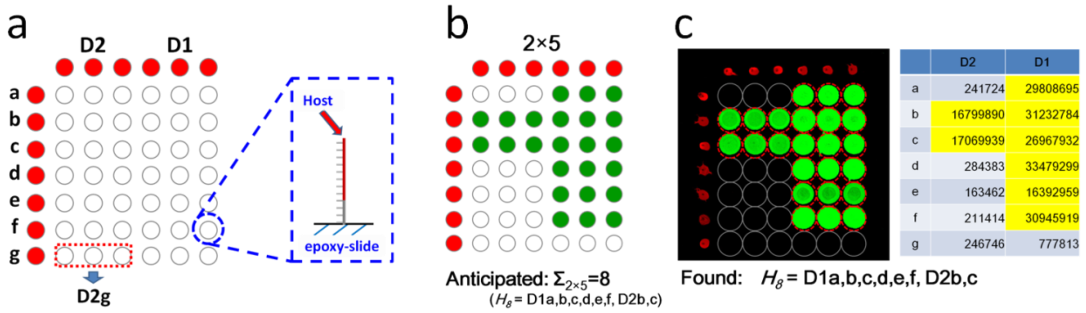

	D2	D1
a	241724	29808695
b	16799890	31232784
c	17069939	26967932
d	284383	33479299
e	163462	16392959
f	211414	30945919
g	246746	777813

Supplementary Figure 11. An array designed for characterizing the correctness of X×Y (X=1,2,3,⋯,9; Y=1,2,3,⋯,9). (a) Principle of the array design: 14 different host strands (Host-D1a,b,c,d,e,f,g and Host-D2a,b,c,d,e,f,g) are printed at their corresponding areas. For clarity, only area D2g is illustrated. Reference horizontal and vertical lines are preprinted with Cy5 dye. This array in principle equals the two digit seven-segmented display used in this work (Fig. 5a and Supplementary Fig. 6) but with different spatial layout of host strands. Therefore it could be regarded as a simplified version of the two digit seven-segmented display. (b) An example of “2×5” is used to explain the criterion used for determining the correctness. For “2×5”, areas D1a,b,c,d,e,f and D2b,c should have positive signals and therefore the anticipated number of positive areas ($\Sigma_{2\times5}$) is 8. (c) The experimental readout of “2×5”. Firstly, 8 areas with the highest signal intensities are chosen from the intensity table (right). Secondly, the found 8 areas are compared with the anticipated 8 areas. If there is fully agreement between these two groups, then the readout is considered to be correct.

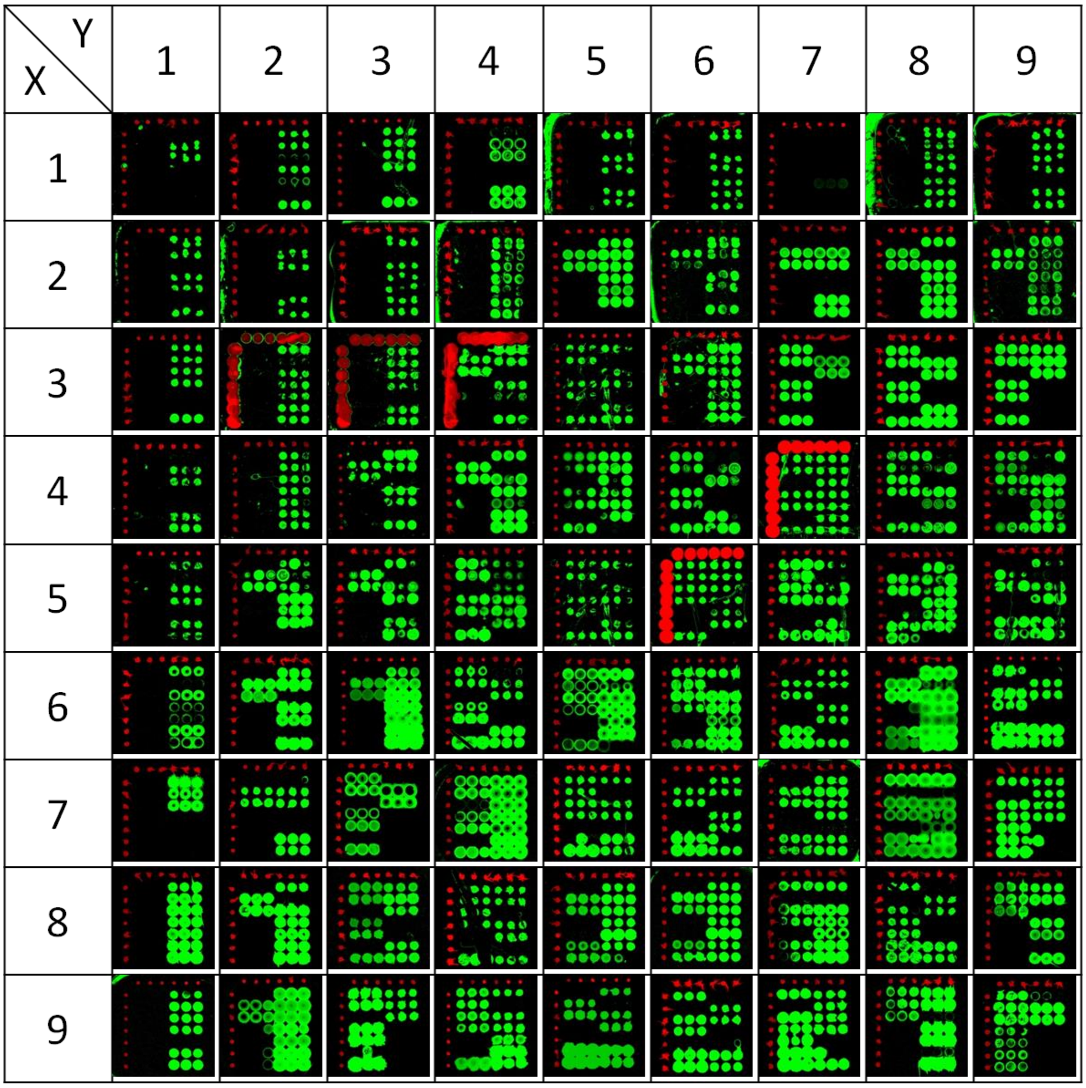

Supplementary Figure 12. The readouts of X×Y (X=1,2,3,···,9; Y=1,2,3,···,9) showing on the host array used for characterizing the correctness.

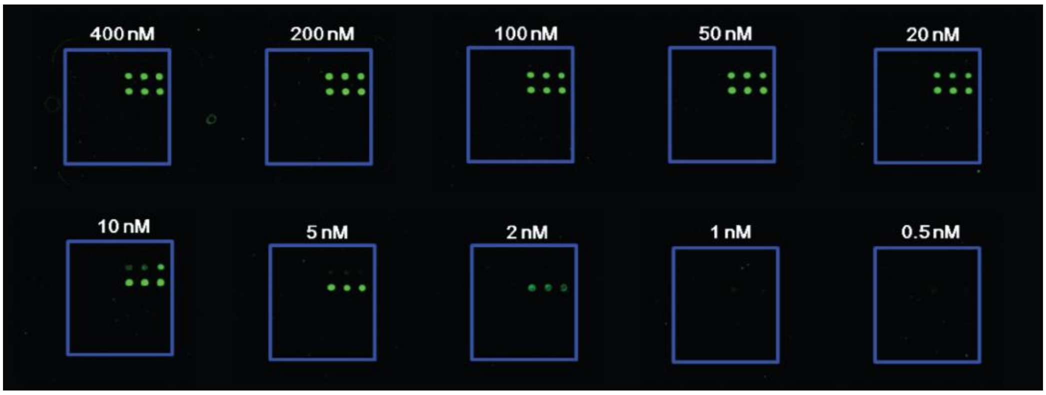

Supplementary Figure 13. Limit of detection using the result complex from the calculation 1×1 at concentration from 0.5 nM to 400 nM of the result complex (As shown in Figure 5B). The limit of detection was determined to be 10 nM.

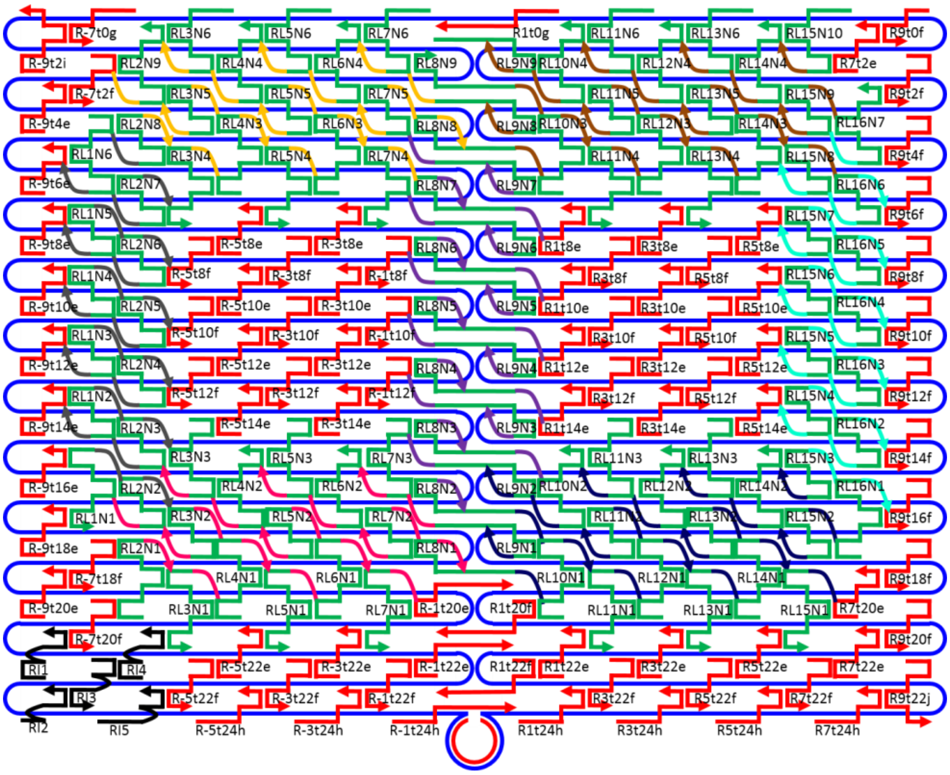

Supplementary Figure 14. Structure of the one-digit DNA origami display.

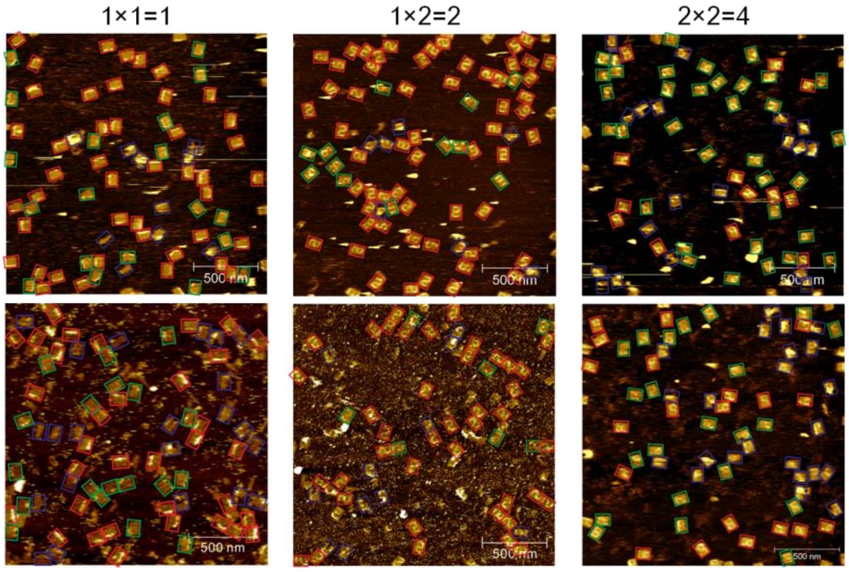

X×Y	correct	uncertain	not well-formed	Number of origami
1×1=1	52.7%	25.7%	21.6%	148
1×2=2	72.3%	14.6%	13.1%	137
2×2=4	25.0%	42.4%	32.6%	132

Supplementary Figure 15. Counted yields of the three calculations showing on DNA origami.

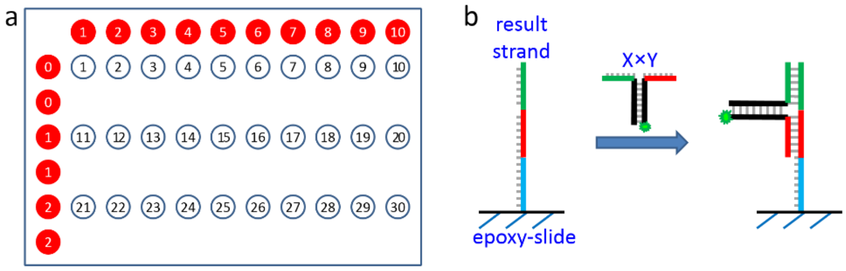

c

position	result strands
1	result11
2	result12, 21
3	result13, 31
4	result14, 22
5	result15
6	result23, 32
8	result24
9	result33
10	result25
12	result34
15	result35

Supplementary Figure 16. A simplified design for directly reading of the calculated result shown in an array representing sequential numbers. (a) The design of the chip. Each number shown in black refers to a specific calculated result. (b) The mechanism for reading. Specific result strands are printed on their corresponding positions and could capture their corresponding fluorescence-labelled X×Y. The fluorescent signal could be read out on a certain position which means the value of the result. (c) The list of the result strands that should be printed on each position.

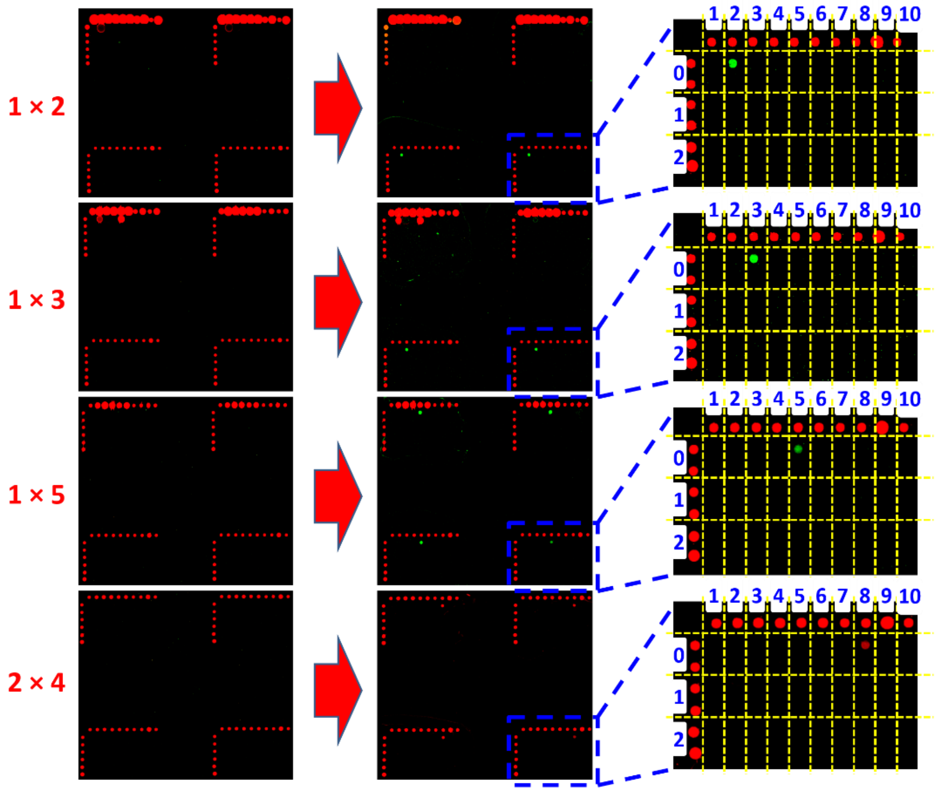

Supplementary Figure 17. Some multiplications based on the simplified design. For 1×2, 1×3 and 1×5, Cy3-labelled input X was used; for 2×4, Cy5-labelled input X was used.

Supplementary Table 1: The accuracy of computations. X×Y highlighted with yellow is determined as correct and highlighted with red is false.

X×Y	Σ	anticipated	found	X×Y	Σ	anticipated	found	X×Y	Σ	anticipated	found
1×1	2	D1b,c	D1b,c	2×1	2	D1a,b,d,e,g	D1a,b,d,e,g	3×1	5	D1a,b,c,d,g	D1a,b,c,d,g
1×2	5	D1a,b,d,e,g	D1a,b,d,e,g	2×2	4	D1b,c,f,g	D1b,c,f,g	3×2	6	D1a,c,d,e,f,g	D1a,c,d,e,f,g
1×3	5	D1a,b,c,d,g	D1a,b,c,d,g	2×3	6	D1a,c,d,e,f,g	D1a,c,d,e,f,g	3×3	6	D1a,b,c,d,f,g	D1a,b,c,d,f,g
1×4	4	D1b,c,f,g	D1b,c,f,g	2×4	7	D1a,b,c,d,e,f,g	D1a,b,c,d,e,f,g	3×4	7	D1a,b,d,e,g; D2b,c	D1a,b,d,e,g; D2b,c
1×5	5	D1a,c,d,f,g	D1a,c,d,f,g	2×5	8	D1a,b,c,d,e,f; D2b,c	D1a,b,c,d,e,f; D2b,c	3×5	7	D1a,c,d,f,g; D2b,c	D1a,c,d,f,g; D2b,c
1×6	6	D1a,c,d,e,f,g	D1a,c,d,e,f,g	2×6	7	D1a,b,d,e,g; D2b,c	D1a,b,d,e,g; D2b,c	3×6	9	D1a,b,c,d,e,f,g; D2b,c	D1a,b,c,d,e,f,g; D2b,c
1×7	3	D1a,b,c	D1c,e,f	2×7	6	D1b,c,f,g; D2b,c	D1b,c,f,g; D2b,c	3×7	7	D1b,c; D2a,b,d,e,g	D1b,c; D2a,b,d,e,g
1×8	7	D1a,b,c,d,e,f,g	D1a,b,c,d,e,f,g	2×8	8	D1a,c,d,e,f,g; D2b,c	D1a,c,d,e,f,g; D2b,c	3×8	9	D1b,c,f,g; D2a,b,d,e,g	D1b,c,f,g; D2a,b,d,e,g
1×9	6	D1a,b,c,d,f,g	D1a,b,c,d,f,g	2×9	9	D1a,b,c,d,e,f,g; D2b,c	D1a,b,c,d,e,f,g; D2b,c	3×9	8	D1a,b,c; D2a,b,d,e,g	D1a,b,c; D2a,b,d,e,g
4×1	4	D1b,c,f,g	D1b,c,f,g	5×1	5	D1a,c,d,f,g	D1a,c,d,f,g	6×1	6	D1a,c,d,e,f,g	D1a,c,d,e,f,g
4×2	7	D1a,b,c,d,e,f,g	D1a,b,c,d,e,f,g	5×2	8	D1a,b,c,d,e,f,g; D2b,c	D1a,b,c,d,e,f,g; D2b,c	6×2	7	D1a,b,d,e,g; D2b,c	D1a,b,d,e,g; D2b,c
4×3	7	D1a,b,d,e,g; D2b,c	D1a,b,d,e,g; D2b,c	5×3	7	D1a,c,d,f,g; D2b,c	D1a,c,d,f,g; D2b,c	6×3	9	D1a,b,c,d,e,f,g; D2b,c	D1a,b,c,d,e,f,g; D2b,c
4×4	8	D1a,c,d,e,f,g; D2b,c	D1a,c,d,e,f,g; D2b,c	5×4	11	D1a,b,c,d,e,f; D2a,b,d,e,g	D1a,b,c,d,e,f; D2a,b,d,e,g	6×4	9	D1b,c,f,g; D2a,b,d,e,g	D1b,c,f,g; D2a,b,d,e,g
4×5	11	D1a,b,c,d,e,f; D2a,b,d,e,g	D1a,b,c,d,e,f; D2a,b,d,e,g	5×5	10	D1a,c,d,f,g; D2a,b,d,e,g	D1a,c,d,f,g; D2a,b,d,e,g	6×5	11	D1a,b,c,d,e,f; D2a,b,c,d,g	D1a,b,c,d,e,f; D2a,b,c,d,g
4×6	9	D1b,c,f,g; D2a,b,d,e,g	D1b,c,f,g; D2a,b,d,e,g	5×6	11	D1a,b,c,d,e,f; D2a,b,c,d,g	D1a,b,c,d,e,f; D2a,b,c,d,g	6×6	11	D1a,c,d,e,f,g; D2a,b,c,d,g	D1a,c,d,e,f,g; D2a,b,c,d,g
4×7	12	D1a,b,c,d,e,f,g; D2a,b,d,e,g	D1a,b,c,d,e,f,g; D2a,b,d,e,g	5×7	10	D1a,c,d,f,g; D2a,b,c,d,g	D1a,c,d,f,g; D2a,b,c,d,g	6×7	9	D1a,b,d,e,g; D2b,c,f,g	D1a,b,d,e,g; D2b,c,f,g
4×8	10	D1a,b,d,e,g; D2a,b,c,d,g	D1a,b,d,e,g; D2a,b,c,d,g	5×8	10	D1a,b,c,d,e,f; D2b,c,f,g	D1a,b,c,d,e,f; D2b,c,f,g	6×8	11	D1a,b,c,d,e,f,g; D2b,c,f,g	D1a,b,c,d,e,f,g; D2b,c,f,g
4×9	11	D1a,c,d,e,f,g; D2a,b,c,d,g	D1a,c,d,e,f,g; D2a,b,c,d,g	5×9	9	D1a,c,d,f,g; D2b,c,f,g	D1a,c,d,f,g; D2b,c,f,g	6×9	9	D1b,c,f,g; D2a,c,d,f,g	D1b,c,f,g; D2a,c,d,f,g
7×1	3	D1a,b,c	D1a,b,c	8×1	7	D1a,b,c,d,e,f,g	D1a,b,c,d,e,f,g	9×1	6	D1a,b,c,d,f,g	D1a,b,c,d,f,g
7×2	6	D1b,c,f,g; D2b,c	D1b,c,f,g; D2b,c	8×2	8	D1a,c,d,e,f,g; D2b,c	D1a,c,d,e,f,g; D2b,c	9×2	9	D1a,b,c,d,e,f,g; D2b,c	D1a,b,c,d,e,f,g; D2b,c
7×3	7	D1b,c; D2a,b,d,e,g	D1b,c; D2a,b,d,e,g	8×3	9	D1b,c,f,g; D2a,b,d,e,g	D1a,b,c,f,g; D2a,b,e,g	9×3	8	D1a,b,c; D2a,b,d,e,g	D1a,b,c; D2a,b,d,e,g
7×4	12	D1a,b,c,d,e,f,g; D2a,b,d,e,g	D1a,b,c,d,e,f,g; D2a,b,d,e,g	8×4	10	D1a,b,d,e,g; D2a,b,c,d,g	D1a,b,d,e,g; D2a,b,c,d,g	9×4	11	D1a,c,d,e,f,g; D2a,b,c,d,g	D1a,c,d,e,f,g; D2a,b,c,d,g
7×5	10	D1a,c,d,f,g; D2a,b,c,d,g	D1a,c,d,f,g; D2a,b,c,d,g	8×5	10	D1a,b,c,d,e,f; D2b,c,f,g	D1a,b,c,d,e,f,g; D2b,c,g	9×5	9	D1a,c,d,f,g; D2b,c,f,g	D1a,c,d,f,g; D2b,c,f,g
7×6	9	D1a,b,d,e,g; D2b,c,f,g	D1a,b,d,e,g; D2b,c,f,g	8×6	11	D1a,b,c,d,e,f,g; D2b,c,f,g	D1a,b,c,d,e,f,g; D2b,c,f,g	9×6	9	D1b,c,f,g; D2a,c,d,f,g	D1b,c,f,g; D2a,c,d,f,g
7×7	10	D1a,b,c,d,f,g; D2b,c,f,g	D1a,b,c,d,f,g; D2b,c,f,g	8×7	11	D1a,c,d,e,f,c; D2a,c,d,f,g	D1a,c,d,e,f,c; D2a,c,d,f,g	9×7	11	D1a,b,c,d,g; D2a,c,d,e,f,g	D1a,b,c,d,g; D2a,c,d,e,f,g
7×8	11	D1a,c,d,e,f,c; D2a,c,d,f,g	D1a,c,d,e,f,c; D2a,c,d,f,g	8×8	10	D1b,c,f,g; D2a,c,d,e,f,g	D1b,c,f,g; D2a,c,d,e,f,g	9×8	8	D1a,b,d,e,g; D2a,b,c	D1a,b,d,e,g; D2a,b,c
7×9	11	D1a,b,c,d,g; D2a,c,d,e,f,g	D1a,b,c,d,f; D2a,c,d,e,f,g	8×9	8	D1a,b,d,e,g; D2a,b,c	D1a,b,d,e,g; D2a,b,c	9×9	9	D1b,c; D2a,b,c,d,e,f,g	D1b,c; D2a,b,c,d,e,f,g
